# Long‐term outcomes of patients undergoing coronary sinus reducer implantation ‐ A multicenter study

**DOI:** 10.1002/clc.23566

**Published:** 2021-02-19

**Authors:** Maayan Konigstein, Francesco Ponticelli, Carlo Zivelonghi, Ilan Merdler, Miri Revivo, Stefan Verheye, Francesco Giannini, Shmuel Banai

**Affiliations:** ^1^ Tel Aviv Medical Center and Sackler School of Medicine Tel Aviv University Tel Aviv‐Yafo Israel; ^2^ Cardiovascular Center Interventional Cardiology Unit, GVM Care & Research Maria Cecilia Hospital Cotignola Italy; ^3^ Division of Heart Center ZNA Middelheim Hospital, Lindendreef Antwerp Antwerp Belgium

**Keywords:** angina, coronary sinus, reducer, refractory angina

## Abstract

**Background:**

Coronary sinus (CS) narrowing by reducer implantation has emerged as a safe and effective therapy for patients suffering from refractory angina. However, data regarding the clinical benefit of this treatment over time is lacking.

**Methods:**

Patients undergoing successful reducer implantation were enrolled prospectively to clinical registries at three medical centers. Those with more than 2‐years of follow‐up were included in the present analysis. Peri‐procedural data, data regarding adverse events, and current evaluation of angina severity (Canadian Cardiovascular Society [CCS] class) were collected.

**Results:**

Overall, 99 consecutive patients (77% males, mean age 69.8 ± 9.4) with severe angina were enrolled between September 2010 and October 2017 and included in the present analysis. No procedure‐related complications were recorded. During a median follow up time of 3.38 years (IQR 2.95–4.40), 15.1% of the patients died, 9% experienced myocardial infarction (MI) and 21% underwent percutaneous coronary intervention (PCI). Mean CCS class was 3.1 ± 0.5 at baseline, improved to 1.66 ± 0.8 at 1 year (p < .001), and remained low through 2‐years and at last follow up (1.72 ± 0.8 and 1.71 ± 0.8, p > 0.5 for both, in comparison to 1 year). At baseline 91% of patients reported severe disabling angina (CCS class 3–4), at 1 year only 17.9% suffered from disabling angina, p < .001, and this portion remained low overtime (19% at last follow up).

**Conclusion:**

Long‐term mortality of patients undergoing reducer implantation is similar to that reported for patients with stable coronary artery disease. The previously reported short‐term efficacy of the reducer, reflected by significant improvement of angina symptoms, is maintained over time.

## 1 INTRODUCTION

Angina, refractory to medical and interventional therapies is a disabling condition and a major public health problem that affects millions of people worldwide. It is common not only in patients with obstructive coronary artery disease with no option for revascularization, but also in 20–40% of patients following successful revascularization.^1^, ^2^, ^3^, ^4^ Refractory angina may also be a symptom of a wide range of other clinical entities, including microvascular disease, hypertrophic cardiomyopathy, and left ventricular diastolic dysfunction.

Coronary Sinus (CS) narrowing by reducer implantation has emerged as a safe and effective therapy for patients suffering from severe refractory angina who are not good candidates for revascularization.^5^, ^6^ The reducer is a stainless‐steel mesh designed to create a fixed focal narrowing in the CS thus increasing backwards pressure, which leads to redistribution of blood from the less ischaemic subepicardium layers to the more ischemic subendocardium, with a consequent symptoms relief.^5^, ^7^

Several clinical reports described the feasibility, safety and efficacy of CS narrowing.^8^, ^9^, ^10^, ^11^, ^12^, ^13^, ^14^, ^15^ Moreover, a prospective, randomized, double‐blind, sham‐controlled clinical trial (the COSIRA trial) demonstrated a significant improvement in symptoms among patients with severe refractory angina (CCS class 3–4) treated with CS narrowing compared with sham‐treated patients, despite, and on top of a strong placebo effect.^16^ Nevertheless, data regarding long term (>2 years) outcomes following reducer implantation is lacking.

The purpose of the present study was to explore the long‐term (>2 years) outcomes of refractory angina patients treated with CS reducer implantation.

## 2 METHODS

This prospective, single arm, registry study includes 99 consecutive patients who underwent reducer implantation between September 2010 and October 2017. This study includes the consolidated experience with the reducer implantation procedure in three high‐volume medical centers (Tel‐Aviv Medical Center, Tel‐Aviv, Israel; San Raffaele Hospital, Milan, Italy; Cardiovascular Center, Ziekenhuis Netwerk Antwerpen Middelheim, Antwerp, Belgium). In order to report exclusively long term outcomes, only data from patients who completed at least 2‐years of follow‐up since the procedure were included.

All patients had obstructive coronary artery disease (CAD) and chronic angina pectoris (Canadian Cardiovascular Society [CCS] classes 2–4) despite maximally tolerated medical therapy, and were considered not amenable to further percutaneous or surgical revascularization procedures. Pre‐implant objective demonstration of ischemia with either treadmill/ pharmacologic stress test, myocardial stress scintigraphy, stress echocardiography or myocardial magnetic resonance was mandatory.

All patients provided written informed consent to undergo the procedure and to participate in the clinical study that was approved by the IRB and ethics committee at each participating center.

The reducer device has been previously described in detail in the literature.^5^, ^7^, ^9^ Briefly, it is a stainless‐steel balloon expandable, hourglass shaped mesh, designed to be introduced into the CS through the internal jugular vein. Several weeks following implantation, the reducer is covered with tissue, and only then, when the distal and proximal metal struts are covered, CS narrowing is established, and the pressure gradient is generated. The diameter at the narrowed mid portion of the reducer is 3 mm, and the distal and proximal ends can reach diameters of 7–13 mm using inflation pressures of two to four bars.

The technical aspects of the procedure were previously reported.^5^, ^7^, ^9^ In short, following pre‐treatment with aspirin and clopidogrel, under local anesthesia, a 6F diagnostic multi‐purpose catheter is introduced into the CS through a 9F introducer sheath in the right or left internal jugular vein. Following angiography of the CS, the optimal site for implantation is determined according to the CS diameter and to avoid side branch bifurcation. The reducer, crimped on a balloon, is introduced over the wire in a 9F guiding catheter into the CS, positioned at the desired site, and implanted by inflating the delivery balloon to achieve slight over‐sizing. Post implantation angiography is performed to ensure appropriate implantation, patency, and appropriate reduction of the lumen's diameter.

Procedural data, in‐hospital outcomes and follow‐up data were recorded. Patients were evaluated at 6 months and 1 year following the procedure as described in previous reports.^10^, ^12^ Long‐term data were collected from medical documents, and by personal interviews. Data regarding cardiovascular events, hospitalizations, repeat coronary angiographies and PCIs were recorded, along with evaluation of patient's CCS class at the time of the clinical interview (CCS 1 represents angina only with strenuous exertion, while CCS 4 represents angina at minimal effort and at rest). Data regarding mortality were extracted from national health registries.

Categorical variables were reported as numbers or percentages. Continuous variables were tested for normal distribution using histograms and Q‐Q Plots, and for convenience purposes continious and ordinal variables were reported as means and standard deviations. CCS class was considered as an ordinal variable, and non parametric tests were used for its analysis. A p value of <0.05 was considered significant for all analyses. All statistical analyses were performed using IBM SPSS Statistics Software for Windows, Version 25.0 (Armonk, NY: IBM Corp).

## 3 RESULTS

Original population of patients undergoing successful reducer implantation at the participating centers consisted of 197 patients. Of those, 45 patients were excluded because they underwent the procedure less than 2 years prior to the study, 16 patients did not survive to 2 years and 33 patients were lost to clinical long term (>2 years) follow up. Therefore, final study cohort included 99 patients (77% males, mean age 69.8 ± 9.4) with severe angina that were enrolled between September 2010 and October 2017. Mean follow up time was 3.9 ± 1.5, (median 3.38 [IQR 2.95–4.40]). Baseline clinical characteristics and procedural data are presented in Table 1. Study population is characterized by high prevalence of cardiovascular risk factors and prior PCI and coronary artery bypass grafting (CABG) in 83.3% and 78.8% of patients, respectively. Access site was the right jugular vein in 96 (97%) patients, and there were no procedural related complications, except one case of device migration. In that case, the reducer was retrieved by snaring through the femoral vein and another device was successfully implanted without any adverse consequences.

**TABLE 1:** Baseline clinical characteristics and medical therapy of the study population (*n* = 99)

Clinical characteristics	
Age, years (mean ± SD)	69.8 ± 9.4
Male gender	76 (76.8%)
Diabetes mellitus	44 (44.4%)
Hypertension	82(82.8%)
Hypercholesterolemia	97 (98.0%)
Smoking history	44 (44.2%)
Family history of ischemic heart disease	54 (54.5%)
Previous stroke/TIA^a^	11 (11.1%)
Previous MI^b^	51 (51.5%)
Previous PCI^c^	83 (83.3%)
Previous CABG^d^	78 (78.8%)
Atrial fibrillation	7 (7.1%)
COPD^e^	15 (15.2%)
Pacemaker	6 (6.1%)
CCS^f^ class	
I	0
II	9 (9.1%)
III	72 (72.7%)
IV	18 (18.2%)
Mean CCS class (mean ± SD)	3.1 ± 0.5
Antianginal therapy	
Beta blocker	81 (81.8%)
Calcium channel blocker	43 (43.4%)
Nitrates	61 (61.6%)
Ivabradine	19 (19.2%)
Ranolazine	25 (25.3%)
reducer implantation Procedural data	
Access (right internal jugular)	96 (97%)
Right atrial pressure (mean ± SD)	4.85 ± 2.5
Access site complication	0 (0%)
Device embolization	1 (1%)
Cardiac tamponade	0 (0%)
Peri‐procedural death	0 (0%)

^a^ transient ischemic attack,

^b^ myocardial infarction,

^c^ percutaneous coronary intervention,

^d^ coronary arterial bypass grafting,

^e^ chronic obstructive pulmonary disease,

^f^ Canadian Cardiovascular Society class.

Long‐term clinical outcomes are presented in Table 2. During a median follow up of 3.38 years, total mortality rate was 15.1%. Importantly, as the study population included only patients who survived and completed at least 2‐years of follow‐up, in order to report the accurate mortality rate of patients undergoing this procedure, the authors performed another analysis to also include patients which were enrolled in the clinical study but did not reach 2‐years of follow up. Mortality rate among this population (*n* = 197) was 15.7% (31/197) with a mean time to death of 3.2 ± 2.3 years.

**TABLE 2:** Long term (median 3.38 [IQR 2.95–4.40]) clinical outcomes (*n* = 99)

Outcome	N of patients (%)
Mortality	15 (15.1)
MI^a^	9 (9.0)
Stroke	3 (3.0)
Repeat angiography	31 (31.3)
Repeat PCI^b^	21 (21.2)
Hospitalization d/t angina	28 (28.2)

^a^ myocardial infarction.

^b^ percutaneous coronary intervention.

Overall, 9% of patients experienced myocardial infarction, and 31.3% and 21.2% of patients underwent coronary angiography and PCI respectively, during follow‐up.

**FIGURE 1:**
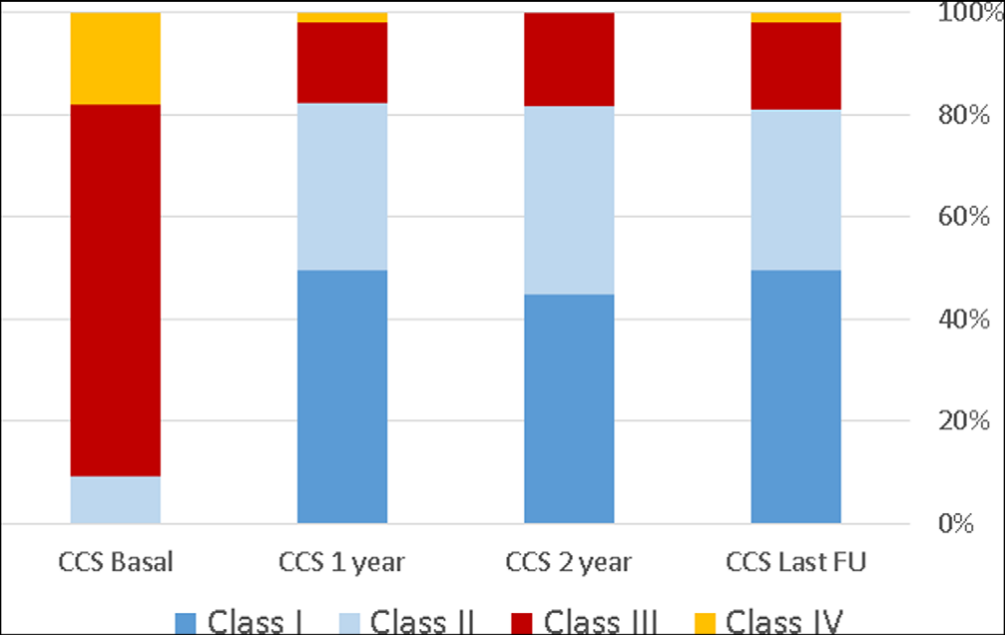
CCS class over time, distribution of CCS among study population at baseline and during follow up. CCS, Canadian cardiovascular society

Mean CCS class improved from 3.1 ± 0.5 at baseline, to 1.66 ± 0.8 in1 year (p < .001), and remained similar in 2‐years and throughout the entire follow‐up to the last follow up visit (1.72 ± 0.8 and 1.71 ± 0.8, p = 0.53 and 0.86 [in comparison to 1 year], respectively, (Table 3)). The distribution of CCS class among the study population during the follow up period is presented in Table 3 and Figure 1. At baseline 91% of patients reported severe disabling angina at rest and minimal effort (CCS class 3–4). At 1‐year follow‐up, the rate of patients suffering from severe disabling angina was reduced to 17.9%, p < .001, and this low rate remained unchanged at the last follow up visit.

**TABLE 3:** Distribution of CCS class at baseline and during follow up (*n* = 99)

CCS class	CCS Baseline	CCS 1 year	CCS 2 year	CCS Last FU (median 3.38, IQR 2.95–4.40)
Mean	3.1 ± 0.5	1.66 ± 0.8	1.72 ± 0.8	1.71 ± 0.8
Class I	0%	49.5%	44.7%	49.5%
Class II	9.1%	32.6%	36.8%	31.6%
Class III	72.7%	15.8%	18.4%	16.8%
Class IV	18.2%	2.1%	0%	2.1%

Abbreviation: CCS, Canadian cardiovascular society.

## 4 DISCUSSION

This is the first study to investigate the long term outcomes (>2 years) of patients treated with CS reducer for refractory angina. The main findings of the study can be summarized as follows: First, reducer implantation is safe, as there were no device or procedure related adverse events, and long‐term mortality, which was ~15% in a mean follow up of 4 years, is similar to what was previously reported for patients with stable ischemic heart disease.^17^ Second, clinical benefit of angina relief, as reflected by a significant reduction of CCS class was maintained over the long‐term follow‐up. Moreover, the proportion of the severely disabled “no‐option” patients (CCS class 3–4) decreased from 91% at baseline to 18% at 1‐year, and remained low during long‐term follow up. Of note, the magnitude of reduction in CCS class presented here at 1 year is somewhat higher compared to that achieved in the treatment arm of the COSIRA study at 6 months,^16^ and similar to that shown in the largest to date prospective registry study (the REDUCER1 trial).^15^

Accumulating data from multiple clinical registries^8^, ^9^, ^10^, ^11^, ^12^, ^13^, ^15^, ^18^, ^19^ and one randomized controlled trial^16^ have demonstrated that reducer implantation is a safe and effective therapy for patients with refractory angina who until recently were labeled as “no option” patients. In fact, a multicenter study of 139 patients demonstrated reduction of angina severity with improvement in Seattle Angina Questionnaire (SAQ) scores and 6 min walk test (6MWT), without any adverse events.^10^ reducer implantation was also associated with objective improvement of myocardial ischemia and myocardial performance as demonstrated by perfusion cardiac magnetic resonance and dobutamine echocardiography,^12^, ^20^ and improvement in systolic and diastolic left ventricular function,^21^, ^22^ as well as increase in VO2 max in cardiopulmonary stress test.^23^ The improvement in angina severity, quality of life, and the reduction in ischemic burden was also translated into reduction in healthcare costs.^24^ Finally, very long‐term (12‐years) anatomical integrity and patency of the reducer was confirmed by Parikh, et al. using CT angiography 12 years following implantation in seven patients.^25^ However, data regarding long‐term clinical outcomes are still limited, and the long‐term efficacy of this treatment has not been previously described.

In the present study, the authors presented the longest clinical follow‐up of patients undergoing this novel treatment to date, and showed that the shorter term clinical benefit that has been reported in previous studies is maintained over time. The progressive nature of CAD in this population, which is characterized by severe and diffuse obstructive CAD, is the root cause of the MI and PCI rates observed in this cohort. Overall, ~30% of patients underwent coronary angiography during follow up, and ~ 20% underwent PCI. The authors report a mortality rate of ~15% in a mean follow up of almost 4 years. This mortality rate is consistent with that previously reported by Henry et al. for patients with stable CAD and refractory angina.^17^

The presumed mechanism of action by which the reducer lessens ischemia has been previously described in detail.^5^ In brief, coronary sinus narrowing leads to increased backward pressure in the coronary venous system, with consequent slight dilatation of the diameter of the arterioles that causes a reduction in vascular resistance in the ischemic subendocardial layers of the myocardium. Consequently, blood flow in the ischemic subendocardium is enhanced, leading to reduction of ischemia severity and extent.

Several limitations should be acknowledged. First, the observational nature of this study precluded us from attenuating the placebo effect, which was widely reported in previous refractory angina studies. However, objective improvement in indices of myocardial ischemia has been demonstrated in previous studies^12^, ^20^ and clinical benefit was already tested in a randomized sham‐controlled study.^16^ Second, as data regarding adverse events were partially collected retrospectively, from clinical documents and patient interviews, it is possible that some events were not captured. Third, differences in data collection and event definitions could exist between centers and might have influenced our results. Fourth, we report the outcomes of patients who completed 2 years of follow up. This methodology might create a survival bias. Therefore, the mortality rate of the entire population (n = 197) is also provided. Finally, data regarding the cause of death were not available for all patients and therefore only total mortality is reported.

In conclusion, in this study, which provides the longest to date clinical follow up of patients undergoing reducer implantation, the benefit of the reducer in alleviating symptoms of angina observed at 6 months – 2 years, is maintained and sustained for longer follow up. The study further establishes this treatment as a valid and beneficial therapeutic option for patients who were until recently considered as “no option” patients. It is still to be investigated, however, in larger long‐term studies, utilizing objective methods of assessment of myocardial ischemia, whether the objective reduction in ischemic burden is also maintained over time.

## CONFLICTS OF INTEREST

Drs. Stefan Verheye and Francesco Giannini serve as proctors for Neovasc Inc. Dr. Shmuel Banai is the Medical Director of Neovasc Inc. All other authors have no conflicts of interest.

## Data Availability

The data that support the findings of this study are available from the corresponding author upon reasonable request
